# Association between Childhood Maltreatment and Suicidal Ideation and Suicide Attempts among Chinese Adolescents: The Moderating Role of Depressive Symptoms

**DOI:** 10.3390/ijerph17176025

**Published:** 2020-08-19

**Authors:** Meiqian Gong, Sheng Zhang, Wenyan Li, Wanxin Wang, Ruipeng Wu, Lan Guo, Ciyong Lu

**Affiliations:** Department of Medical Statistics and Epidemiology, School of Public Health, Sun Yat-sen University, Guangzhou 510080, China; gongmq@mail2.sysu.edu.cn (M.G.); zhangsh46@mail2.sysu.edu.cn (S.Z.); liwy23@mail2.sysu.edu.cn (W.L.); wangwanx@mail2.sysu.edu.cn (W.W.); wurp5@mail2.sysu.edu.cn (R.W.)

**Keywords:** suicidal ideation, suicidal attempts, childhood maltreatment, depressive symptoms, adolescents

## Abstract

Suicidal behavior is a major public health concern worldwide and has become the second-leading cause of death among adolescents. The purposes of this study were to investigate the associations between childhood maltreatment and suicidal behavior and to test whether depressive symptoms have moderating effects on these associations. A multistage stratified cluster randomized sampling method was adopted to collect data from 21,019 high school students in Guangdong Province, China. The prevalence of suicidal ideation and suicide attempts among Chinese adolescents were 18.2% and 3.6%, respectively. Physical abuse (adjusted odds ratios (AOR) = 1.35, 95% confidence intervals (CI) = 1.32–1.38), emotional abuse (AOR = 1.26, 95% CI = 1.25–1.28), sexual abuse (AOR = 1.25, 95% CI = 1.21–1.30), physical neglect (AOR = 1.09, 95% CI = 1.08–1.11), and emotional neglect (AOR = 1.08, 95% CI = 1.08–1.09) were all associated with an increased risk of suicidal ideation, and these associations were also found for suicide attempts. According to stratification analyses, physical abuse/emotional abuse/sexual abuse had a stronger effect on suicidal ideation and suicide attempts among students without depressive symptoms than among students with depressive symptoms. Childhood maltreatment was associated with an increased risk of suicidal ideation and suicide attempts in Chinese adolescents. Depressive symptoms play a moderating role in the association between childhood maltreatment and suicidal behaviors.

## 1. Introduction

Suicidal behavior, including suicidal ideation, suicidal attempts, and completed suicide, threatens people’s lives and the quality of life of survivors. More than 800,000 people die every year from suicide worldwide [1], and the sharp increase in suicidal deaths occurs among adolescents aged 15–19 years [2]. Notably, suicide has become the second-leading cause of death among adolescents [3]. According to a study, we learned that the prevalence rate of suicidal behaviors among United States students ranged from 6% to 25% and among Chinese students ranged from 2.7% to 45.1% [4]. Previous research also suggests that suicidal ideation and suicide attempts are predictors of suicide in adolescents [5] and that the timely identification of them can effectively reduce the incidence of completed suicide.

Childhood abuse is a global phenomenon that affects millions of children worldwide [6], and it includes five types: emotional abuse, physical abuse, sexual abuse, emotional neglect, and physical neglect. According to a review that contains 244 publications worldwide and 551 prevalence rates for the different types of childhood abuse, we learned that the overall estimated prevalence was as follows: physical abuse (22.6%), emotional abuse (36.3%), sexual abuse (12.7%), physical neglect (16.3%), and emotional neglect (18.4%) [6]. Compared with Western countries, Chinese parenting styles are more severe and have been generally accepted by Chinese people. A systematic review shows that 26.6% of children under the age of 18 in China suffer from physical abuse, 19.6% suffer from emotional abuse, 8.7% suffer from sexual abuse, and 26.0% suffer from neglect [7]. The interpersonal theory of suicide proposes that adverse experiences such as childhood maltreatment can form a state of adaptation to pain, can reduce the victim’s fear of self-harm, and can eventually contribute to suicidal behavior [8]. A number of studies have suggested that childhood abuse is related to increased risk of suicidal behavior in adolescents [9,10,11].

Moreover, studies have reported that people who experienced childhood abuse have an increased risk of depressive symptoms [12,13,14]. A study has suggested that abused adolescents are at high risk of depressive symptoms [15]. In addition, the relationships between depressive symptoms and suicidal behavior have been extensively studied. Multiple studies have suggested that depressive symptoms promote suicidal behavior in young people [16,17]. To sum up, we can reasonably assume that depressive symptoms may play a moderating role in the association between childhood maltreatment and suicidal behavior among adolescents.

Childhood maltreatment has a far-reaching negative impact on the physical and mental development of individuals [18,19]. However, there are scarce studies on the impact of childhood abuse in China [7]. In addition, although many evidences have shown that childhood abuse is associated with suicidal behavior, the relationship and strength between different types of childhood maltreatment and suicidal behavior varies across countries and populations [20,21]. Moreover, the interaction between childhood abuse, depressive symptoms, and suicidal behavior remains controversial. For example, a study analyzed the interaction between childhood abuse and mental disorders, and its impacts on suicidal behavior and found that childhood abuse had a significant impact on suicidal behavior in the general population but not on individuals with mood disorders [20]. However, another study showed that the interaction terms of various types of childhood maltreatment and depression symptoms were not significantly related to suicide [22]. Finally, few studies have explored the potential moderating role of depressive symptoms in the association between childhood abuse and suicidal behavior in Chinese adolescents. Therefore, the purposes of this study were (1) to investigate the associations as well as the strengths between childhood maltreatment and suicidal behavior and (2) to test whether these associations and strengths vary with adolescents’ depressive symptoms.

## 2. Materials and Methods

### 2.1. Study Design and Participants

This is a cross-sectional study, and the study participants are students in Guangdong Province in the 2018–2019 academic year. A multistage stratified cluster randomized sampling method was adopted. First, the regional economic development across Guangdong is relatively uneven. Therefore, according to the GDP per capita level in 2017, all cities in Guangdong were divided into three groups: high, medium, and low. Two cities were randomly selected from each level as representatives. Second, six junior high schools, four senior high schools, and two vocational high schools were randomly selected in each city. Third, two classes were randomly selected from each grade in each school. All students in the selected classes were invited to participate in the survey. A total of 21,019 students were invited to participate in our study, and 20,517 valid questionnaires were ultimately obtained, with a response rate of 97.6%. Our self-report questionnaire was anonymous, and the students filled out the questionnaires only with the investigators but without the teachers. It took nearly three months to collect all the data.

### 2.2. Ethical Statement

The cross-sectional study of adolescents in Guangdong Province was ethically approved by the Sun Yat-sen University School of Public Health Institutional Review Board (Ethical Approval Number: 2012(20)). After the purpose and significance of the study had been fully explained, written informed consent was obtained from each student (aged 18 or above) or their parents (aged under 18).

### 2.3. Measures

#### 2.3.1. Suicidal Ideation and Suicidal Attempts

The assessment of suicidal ideation and suicide attempts uses the following questions: “During the past year, how many times did you seriously consider attempting suicide?” for suicidal ideation and “During the past year, how many times did you actually attempt suicide?” for suicide attempts. Available answers were “0”, “1”, and “at least 2 times”; choosing “1” or “at least 2 times” represents suicidal ideation/suicide attempts [23].

#### 2.3.2. Childhood Maltreatment

Childhood maltreatment was evaluated using the short form of the Childhood Trauma Questionnaire (CTQ-SF) in Chinese [24,25]. The CTQ-SF has good reliability and validity among the Chinese student population [26] and showed high internal consistency (Cronbach’s alpha = 0.77) [27]. In addition, it has been extensively used in a number of studies on Chinese adolescents [28,29].

The scale contains five subscales to assess five types of childhood maltreatment. Each subscale contains five questions, each question has 5 possible response options, and scores of 1–5 are given according to the selected options. The overall scale had scores ranging from 25 to 125. The higher the scores, the more severe the childhood abuse.

#### 2.3.3. Depressive Symptoms

The Chinese version of the Center for Epidemiology Scale for Depression (CES-D) was used to assess whether the respondents had depression symptoms. This Chinese version of the scale has been verified by scholars and is widely used in Chinese [30,31]. The scale showed high internal consistency among Chinese adolescents (Cronbach’s alpha = 0.88) [32]. This scale contains 20 items about depressive symptoms, and each item has four alternative answers; scores of 0–3 are given according to the selected answers. The total score of the scale is the sum of the scores of each item, with a minimum score of 0 and a maximum score of 60. The higher the scores are, the more severe the depressive symptoms. In this study, a cutoff value of 28 was used to classify whether the subjects had depressive symptoms [33,34].

#### 2.3.4. Covariates

Factors associated with suicide or childhood abuse were taken into consideration [35,36,37]. Demographic factors included age, gender, and grade. Family-related factors included living arrangements, household socioeconomic status (HSS), and parental marital status. School-related factors included academic pressure, classmate relations, and teacher–classmate relations. Living arrangements were assessed by asking students who lived in their primary home (responses included “both biological parents”, “only father or mother”, or “other”). The answers to questions about the HSS (answers included “good”, “average”, or “poor”), parental marital status (answers included “harmonious”, “often quarrel”, or “separated or divorced”), academic pressure (answers included “above average”, “average”, and “below average”), and classmate relations and teacher–classmate relations (answers included “good”, “average”, or “poor”) were all ranked based on the students’ self-perception. Smoking and drinking were assessed by the following question: “Have you smoked at least one cigarette (or drunk alcohol) during your lifetime?” The response options were “yes” or “no”.

### 2.4. Statistical Analysis

First, descriptive analyses for age, sex, childhood abuse, depressive symptoms, and suicidal behaviors were conducted. Continuous variables were described as the mean and standard deviation (SD), while categorical variables were expressed as the number and percentage (%). Chi-square tests for categorical variables and t-tests and for continuous variables were used to compare the differences between groups.

Second, univariate logistic regression models were used to assess the association between different types of childhood maltreatment and depressive symptoms with suicidal ideation or suicide attempts. Then, multivariable logistic regression models that adjusted for variables that were significant at the 0.10 level in the univariate analysis or that were extensively reported in the literature were conducted to examine the independent relationship between childhood maltreatment and depressive symptoms with suicidal ideation or suicide attempts, and the adjusted odds ratios (AORs) and 95% confidence intervals (CIs) were acquired.

Third, multivariable logistic regression models were used to test the interaction items between childhood maltreatment and depressive symptoms. If the association between the interaction item and suicidal ideation or suicide attempts was statistically significant, then the analyses stratified by depressive symptoms would be conducted to assess the associations of childhood maltreatment with suicidal ideation and suicide attempts. All statistical tests were two-sided, and the significance level was set at *p* < 0.05. All statistical analyses were conducted using IBM SPSS Statistics 25.0 (IBM, Armonk, NY, USA).

## 3. Results

### 3.1. Sample Characteristics of 20,517 Students

A total of 20,517 students aged 11–20 were included in the analysis, of which 49.8% (10,221/20,517) were female students and 50.2% (10,296/20,517) were male students and the mean age was 15.0 (SD: 1.8) years. In all samples, 3732 (18.2%) students had suicidal ideation and 738 (3.6%) had suicidal attempts in the past year. In addition, at the cutoff value of 28 on the CES-D scale, we detected that 11.0% of the students had depressive symptoms. Moreover, among students with suicidal ideation or suicidal attempts, the scores for all types of childhood maltreatment were higher than those of students without suicidal ideation or suicidal attempts (*p* < 0.001). These results are presented in Table 1.

### 3.2. Association of Childhood Maltreatment and Depressive Symptoms with Suicidality

In the unadjusted model, all five types of childhood abuse and depressive symptoms were significantly associated with suicidal ideation or suicide attempts (*p* < 0.001) (Model 1 in Table 2). After adjustments were made for covariates, our models showed that physical abuse (AOR = 1.35, 95% CI = 1.32–1.38), emotional abuse (AOR = 1.26, 95% CI = 1.25–1.28), sexual abuse (AOR = 1.25, 95% CI = 1.21–1.30), physical neglect (AOR = 1.09, 95% CI = 1.08–1.11), emotional neglect (AOR = 1.08, 95% CI = 1.08–1.09), and depressive symptoms (AOR = 6.77, 95% CI = 6.07–7.54) were all associated with an increased risk of suicidal ideation. In addition, these associations were also found for suicide attempts (Model 2 in Table 2).

### 3.3. Associations of Interaction Items with Suicidal Ideation and Suicidal Attempts

As shown in Table 3, in our multivariable logistic regression models, the relationship between the interaction items (physical abuse/emotional abuse/sexual abuse and depressive symptoms) and suicidal ideation or suicide attempts was statistically significant (all *p* < 0.001). However, no statistical significance was found in the interaction items of physical neglect/emotional neglect and depressive symptoms with suicidal ideation or suicide attempts. In other words, depressive symptoms moderated the association between abuse (physical/emotional/sexual abuse) and suicidal ideation/suicide attempts, while the moderating effects were not significant in the association between neglect (physical neglect and emotional neglect) and suicidal ideation or suicide attempts.

### 3.4. Associations of Childhood Maltreatment and Suicidality Stratified by Depressive Symptoms

After adjustments were made for the covariates, stratified analyses found that, among students with depressive symptoms, the association between sexual abuse and suicidal ideation (AOR = 1.05, 95% CI = 0.99–1.11, *p* = 0.083) or suicide attempts (AOR = 1.05, 95% CI = 1.00–1.12, *p* = 0.072) was not statistically significant. However, among students without depressive symptoms, sexual abuse was significantly associated with suicidal ideation (AOR = 1.26, 95% CI = 1.21–1.31, *p* < 0.001) and suicide attempts (AOR = 1.24, 95% CI = 1.17–1.31, *p* < 0.001). In addition, both physical abuse and emotional abuse were associated with an increased risk of suicidal behavior among students with and without depressive symptoms. However, the associations between physical abuse/emotional abuse and suicidal behaviors were stronger among students without depressive symptoms than among those with depressive symptoms. The above results are summarized in Table 4.

## 4. Discussion

There is a paucity of large-scale cross-sectional studies to explore whether depressive symptoms moderate the association between childhood abuse and suicidal behavior in Chinese students. Our study found that the average scores for each type of childhood maltreatment were as follows: physical abuse 5.7 (1.6), emotional abuse 6.8 (2.9), sexual abuse 5.2 (1.1), physical neglect 7.2 (2.8), and emotional neglect 8.6 (5.2). The scores for various types of childhood abuse are similar to those found in a 2014–2015 survey among adolescents in Chongqing, and in the above order of childhood abuse, the average scores were 5.6 (1.7), 6.6 (3.0), 5.5 (1.8), 7.8 (3.1), and 8.7 (5.4), respectively [29]. In addition, this study reported that the prevalence of suicidal ideation among adolescents was 18.2% and that the prevalence of suicide attempts was 3.6%. The prevalence of suicidal ideation in the study was similar to that reported by the Youth Risk Behavior Surveillance System (YRBSS) in 2017 (17.2%), but the prevalence of suicide attempts was lower than in the United States (7.4%) [38]. The study also found that the prevalence of both suicidal ideation and suicide attempts was higher in girls than in boys (suicidal ideation: 13.0% for boys, 23.4% for girls; suicide attempts: 2.3% for boys and 4.9% for girls), which was consistent with the findings of many other studies [38,39].

This study analyzed the relationship between childhood abuse and suicidal behaviors and whether, in univariate logistic regression models or multivariable logistics regression models that controlled for covariates, all types of childhood abuse were related to increased risk of suicidality. These results are similar to those of many other studies [9,40,41]. However, our study found that, of the five types of maltreatment, physical abuse was most strongly associated with suicidal behavior, followed by emotional abuse. In contrast, another study argued that emotional abuse was the strongest predictor of suicide attempts, followed by physical abuse [20]. These differences may be related to the cultural background and traditional education methods of China and other countries. There are several possible explanations for the relationship between childhood abuse and suicidality. According to the Schematic Appraisals Model for Suicide (SAMS), adverse events in childhood can lead to increasing feelings of self-defeat and eventually to a choice of suicidality as a means of escape [42,43]. From the perspective of biological mechanisms, a previous study found that people who suffered from childhood abuse had a smaller prefrontal cortex volume [44], which was related to cognitive function [45], while cognitive impairment was related to suicidality [46].

In line with the findings of most studies [16,47,48], our study also found that depressive symptoms contributed to suicidal behavior. In addition, our multivariable logistic regression shows that there are three interaction items (depressive symptoms* physical abuse/emotional abuse/sexual abuse) that were significant in relation to suicidal behaviors. Then, stratified analysis by depressive symptoms, the results indicated that the relation between sexual abuse and suicidal behaviors was found only among students without depressive symptoms. In addition, both physical abuse and emotional abuse were associated with an increased risk of suicidal behavior among participants with or without depressive symptoms. However, the associations between physical abuse/emotional abuse and suicidal behaviors were stronger among participants without depressive symptoms than among those with depressive symptoms. The above results suggest that depressive symptoms may moderate the relations between three types of childhood maltreatment (physical/emotional/sexual abuse) and suicidal behavior. Similarly, previous study has reported that childhood maltreatment has a significant effect on suicidal behavior in the general population but not in people with mood disorders [20]. Another study also proposed that “male depression” may play a moderating role in childhood maltreatment and suicidality [49]. However, the role of depressive symptoms in the association between childhood abuse and suicidality was not consistent in different studies. A study of elderly people over 60 years of age showed that the interaction terms of various types of childhood maltreatment and depression symptoms were not significantly related to suicide [22]. In addition, other studies have suggested that depression symptoms may play a mediating role in childhood abuse and suicidal behavior [28,50], but these studies did not rule out the association between the interaction terms of childhood abuse and depression symptoms, and suicidal behavior before the mediation analysis. Possible explanations for the moderating effect of depressive symptoms in our results are as follows. First, it is well known that depressive symptoms are important risk factors for suicide. Suicidal behavior may be more common in students with depressive symptoms than in those without. However, childhood maltreatment (distal risk factors) may be a more important factor in participants without depressive symptoms because, in participants with depressive symptoms, the effect of depressive symptoms on suicidality may be more important than the effect of childhood maltreatment. Second, after stratification by depressive symptoms, the sample size of individuals with depressive symptoms is relatively small and it may be more difficult to obtain statistically significant results.

Our study found that childhood maltreatment, depressive symptoms, and suicidal behavior remain serious problems among Chinese adolescents and should be of widespread concern. Based on our research results, we have the following suggestions. First, a national long-term monitoring system should be established to monitor suicidal behavior among Chinese adolescents. Second, schools should conduct mental health education regularly, and for students with depressive symptoms, timely identification and counseling should be performed. In addition, we suggested that parent-focused trainings specifically targeted at adolescent suicidal behavior should be carried out in schools. Third, parents should recognize the adverse effects of childhood maltreatment on their children and give them more physical or psychological care. Fourth, physicians should focus on the history of childhood maltreatment and depressive symptoms when treating suicidal adolescents.

There are some limitations in this study. First, the cross-sectional study design limits causal inference. Second, it is difficult to avoid potential bias when using a self-report questionnaire to collect data. Third, the study included only students who were not absent from class that day of the investigation. Fourth, students were only from Guangdong Province, so our findings should be carefully considered when applied to other regions.

## 5. Conclusions

Our study found that childhood maltreatment was associated with an increased risk of suicidal behavior in Chinese adolescents. Depressive symptoms moderate the association between three types of childhood abuse (physical/emotional/sexual abuse) and suicidal behaviors. Among students without depressive symptoms, physical/emotional/sexual abuse had a stronger effect on suicidal ideation and suicidal attempts. Our findings suggest that more attention should be paid to childhood maltreatment, depressive symptoms, and suicidal behaviors in Chinese adolescents and that early detection and intervention may contribute to the healthy development of adolescents.

## Figures and Tables

**Table 1:** Sample characteristics of 20,517 students.

Variable	Total # (%)	Suicidal Ideation			Suicidal Attempts		
		Yes # (%)	No # (%)	*p*-Value *	Yes # (%)	No # (%)	*p*-Value *
Total	20,517 (100)	3732 (18.2)	16,785 (81.8)		738 (3.6)	19,779 (96.4)	
Age, mean (SD)	15.0 (1.8)	14.9 (1.7)	15.0 (1.8)	<0.001	14.7 (1.6)	15.0 (1.8)	<0.001
Sex							
Boy	10,296 (50.2)	1342 (13.0)	8954 (87.0)	<0.001	234 (2.3)	10,062 (97.9)	<0.001
Girl	10,221 (49.8)	2390 (23.4)	7831 (76.6)		504 (4.9)	9717 (95.1)	
Grade							
7th	3487 (17.0)	566 (16.2)	2921 (83.8)	<0.001	110 (3.2)	3377 (96.8)	<0.001
8th	3405 (16.6)	691 (20.3)	2714 (79.7)		163 (4.8)	3242 (95.2)	
9th	3317 (16.2)	646 (19.5)	2671 (80.5)		126 (3.8)	3191 (96.2)	
10th	3798 (18.5)	771 (20.3)	3027 (79.7)		184 (4.8)	3614 (95.2)	
11th	3598 (17.5)	565 (15.7)	3033 (84.3)		91 (2.5)	3507 (97.5)	
12th	2912 (14.2)	493 (16.9)	2419 (83.1)		64 (2.2)	2848 (97.8)	
Living arrangement							
Living with parents	15,325 (74.7)	2576 (16.8)	12,749 (83.2)	<0.001	485 (3.2)	14,840 (96.8)	<0.001
Living with a single parent	2677 (13.0)	674 (25.2)	2003 (74.8)		151 (5.6)	2526 (94.4)	
Living with others	2358 (11.5)	456 (19.3)	1902 (80.7)		96 (4.1)	2262 (95.9)	
Missing data	157 (0.8)	NA	NA		NA	NA	
HSS							
Above average	6172 (30.1)	941 (15.2)	5231 (84.8)	<0.001	179 (2.9)	5993 (97.1)	<0.001
Average	11,603 (56.6)	2135 (18.4)	9468 (81.6)		430 (3.7)	11,173 (96.3)	
Below average	2609 (12.7)	636 (24.4)	1973 (75.6)		121 (4.6)	2488 (95.4)	
Missing data	133 (0.6)	NA	NA		NA	NA	
Academic pressure							
Below average	4413 (21.5)	515 (11.7)	3898 (88.3)	<0.001	110 (2.5)	4303 (97.5)	<0.001
Average	9318 (45.4)	1377 (14.8)	7941 (85.2)		273 (2.9)	9045 (97.1)	
Above average	6653 (32.4)	1815 (27.3)	4838 (72.7)		350 (5.3)	6303 (94.7)	
Missing data	133 (0.6)	NA	NA		NA	NA	
Parental marital status							
Harmonious	18,038 (87.9)	2940 (16.3)	15,098 (83.7)	<0.001	553 (3.1)	17,485 (96.9)	<0.001
Often quarrel	586 (2.9)	265 (45.2)	321 (54.8)		69 (11.8)	517 (88.2)	
Separated or divorced	1745 (8.5)	506 (29.0)	1239 (71.0)		114 (6.5)	1631 (93.5)	
Missing data	148 (0.7)	NA	NA		NA	NA	
Classmate relations							
Good	16,699 (81.4)	2633 (15.8)	14,066 (84.2)	<0.001	455 (2.7)	16,244 (97.3)	<0.001
Average	3391 (16.5)	944 (27.8)	2447 (72.2)		234 (6.9)	3157 (93.1)	
Poor	282 (1.4)	124 (44.0)	158 (56.0)		42 (14.9)	240 (85.1)	
Missing data	145 (0.7)	NA	NA		NA	NA	
Teacher-classmate relations							
Good	14,179 (69.2)	2058 (14.5)	12,139 (85.5)	<0.001	369 (2.6)	13,828 (97.4)	<0.001
Average	5716 (27.9)	1482 (25.9)	4234 (74.1)		311 (5.4)	5405 (94.6)	
Poor	376 (1.8)	148 (39.4)	228 (60.6)		49 (13.0)	327 (87.0)	
Missing data	228 (1.1)	NA	NA		NA	NA	
Smoking							
No	18,481 (90.1)	3113 (16.8)	15,368 (83.2)	<0.001	564 (3.1)	17,917 (96.9)	<0.001
Yes	1753 (8.5)	555 (31.7)	1198 (68.3)		153 (8.7)	1600 (91.3)	
Missing data	283 (1.4)	NA	NA		NA	NA	
Drinking							
No	11,832 (57.7)	1472 (12.4)	10,360 (87.6)	<0.001	242 (2.0)	11,590 (98.0)	<0.001
Yes	8379 (40.8)	2202 (26.3)	6177 (73.7)		481 (5.7)	7898 (94.3)	
Missing data	306 (1.5)	NA	NA		NA	NA	
Depressive symptoms							
No	18,269 (89.0)	2361 (12.9)	15,908 (87.1)	<0.001	346 (1.9)	17,923 (98.1)	<0.001
Yes	2248 (11.0)	1371 (61.0)	877 (39.0)		392 (17.4)	1856 (82.6)	
CTQ scores of physical abuse ^a^	5.7 (1.6)	6.6 (2.4)	5.5 (1.2)	<0.001	7.4 (3.3)	5.6 (1.4)	<0.001
CTQ scores of emotional abuse ^a^	6.8 (2.9)	9.3 (4.2)	6.3 (2.2)	<0.001	11.1 (5.2)	6.7 (2.7)	<0.001
CTQ scores of sexual abuse ^a^	5.2 (1.1)	5.6 (1.6)	5.2 (0.9)	<0.001	5.8 (2.2)	5.2 (1.0)	<0.001
CTQ scores of physical neglect ^a^	7.2 (2.8)	8.1 (3.2)	7.0 (2.7)	<0.001	9.0 (3.7)	7.2 (2.8)	<0.001
CTQ scores of emotional neglect ^a^	8.6 (5.2)	11.1 (5.9)	8.1 (4.9)	<0.001	13.4 (6.6)	8.4 (5.1)	<0.001

Abbreviations: HSS, household socioeconomic status; CTQ, childhood trauma questionnaire; NA, not applicable or no data available; SD, standard deviation. # Mean number. ^a^ Data were presented as the mean (SD). * Chi-squared tests were used for categorical variables, and *t* tests were used for age data and the CTQ scores data.

**Table 2:** Association of childhood maltreatment and depressive symptoms with suicidality.

Variable	Models 1				Models 2			
	Suicidal Ideation		Suicidal Attempts		Suicidal Ideation		Suicidal Attempts	
	OR (95% CI)	*p*-Value	OR (95%CI)	*p*-Value	AOR (95% CI)	*p*-Value	AOR (95% CI)	*p*-Value
Physical abuse *	1.46 (1.43–1.50)	<0.001	1.37 (1.33–1.41)	<0.001	1.35 (1.32–1.38)	<0.001	1.28 (1.24–1.32)	<0.001
Emotional abuse *	1.34 (1.32–1.35)	<0.001	1.28 (1.26–1.30)	<0.001	1.26 (1.25–1.28)	<0.001	1.22 (1.20–1.24)	<0.001
Sexual abuse *	1.35 (1.30–1.40)	<0.001	1.23 (1.19–1.28)	<0.001	1.25 (1.21–1.30)	<0.001	1.18 (1.14–1.23)	<0.001
Physical neglect *	1.12 (1.11–1.14)	<0.001	1.20 (1.18–1.23)	<0.001	1.09 (1.08–1.11)	<0.001	1.15 (1.12–1.17)	<0.001
Emotional neglect *	1.10 (1.09–1.11)	<0.001	1.13 (1.12–1.14)	<0.001	1.08 (1.08–1.09)	<0.001	1.11 (1.09–1.12)	<0.001
Depressive symptoms								
No	1.00		1.00		1.00		1.00	
Yes	10.53 (9.58–11.58)	<0.001	10.94 (9.40–12.74)	<0.001	6.77 (6.07–7.54)	<0.001	6.56 (5.47–7.86)	<0.001

Abbreviations: OR, odds ratio; CI, confidence interval; AOR, adjusted odds ratio. Model 1: univariate logistic regression, not adjusted for covariates. Model 2: adjusted for age, grade, gender, living arrangements, household socioeconomic status, academic pressure, parental marital status, relationships with classmates, relationships with teachers, smoking, and drinking. * For every 1 score increase in physical abuse, emotional abuse, sexual abuse, physical neglect, and emotional neglect.

**Table 3:** Associations of interaction items with suicidal ideation and suicidal attempts.

Interaction Item	Suicidal Ideation		Suicidal Attempts	
	AOR (95% CI)	*p*-Value	AOR (95% CI)	*p*-Value
Depressive symptoms ^a^				
Physical abuse	0.82 (0.77–0.86)	<0.001	0.87 (0.82–0.93)	<0.001
Emotional abuse	0.90 (0.87–0.93)	<0.001	0.92 (0.89–0.95)	<0.001
Sexual abuse	0.84 (0.78–0.90)	<0.001	0.84 (0.78–0.91)	<0.001
Physical neglect	0.98 (0.95–1.01)	0.161	0.95 (0.91–1.00)	0.053
Emotional neglect	0.99 (0.97–1.00)	0.115	0.98 (0.95–1.00)	0.087

Abbreviations: AOR, adjusted odds ratio; CI, confidence interval. ^a^ Each type of maltreatment, depressive symptom, and the interaction item between childhood maltreatment and depressive symptoms as well as age, grade, gender, living arrangements, household socioeconomic status, academic pressure, parental marital status, relationships with classmates, relationships with teachers, smoking, and drinking were simultaneously entered into the multivariable logistic regression models.

**Table 4:** Associations of childhood maltreatment and suicidality stratified by depressive symptoms.

Variable	With Depressive Symptoms				Without Depressive Symptoms			
	Suicidal Ideation		Suicidal Attempts		Suicidal Ideation		Suicidal Attempts	
	AOR (95% CI)	*p*-Value	AOR (95% CI)	*p*-Value	AOR (95% CI)	*p*-Value	AOR (95% CI)	*p*-Value
Physical abuse *	1.12 (1.07–1.16)	<0.001	1.15 (1.11–1.20)	<0.001	1.37 (1.33–1.42)	<0.001	1.29 (1.23–1.35)	<0.001
Emotional abuse *	1.13 (1.10–1.16)	<0.001	1.13 (1.10–1.16)	<0.001	1.25 (1.23–1.27)	<0.001	1.22 (1.18–1.25)	<0.001
Sexual abuse *	1.05 (0.99–1.11)	0.083	1.05 (1.00–1.12)	0.072	1.26 (1.21–1.31)	<0.001	1.24 (1.17–1.31)	<0.001
Physical neglect *	1.05 (1.02–1.08)	0.001	1.10 (1.06–1.13)	<0.001	1.07 (1.06–1.09)	<0.001	1.13 (1.09–1.18)	<0.001
Emotional neglect *	1.05 (1.04–1.07)	<0.001	1.07 (1.05–1.09)	<0.001	1.07 (1.06–1.08)	<0.001	1.09 (1.08–1.11)	<0.001

Abbreviations: OR, odds ratio; CI, confidence interval; AOR, adjusted odds ratio. * For every 1 score increase in physical abuse, emotional abuse, sexual abuse, physical neglect, and emotional neglect. Note: All the models were adjusted for age, grade, gender, living arrangements, household socioeconomic status, academic pressure, parental marital status, relationships with classmates, relationships with teachers, smoking, and drinking.
